# Food insecurity and health-related quality of life among a nationally representative sample of older adults: cross-sectional analysis

**DOI:** 10.1186/s12877-024-04716-9

**Published:** 2024-02-01

**Authors:** Abeer A. Aljahdali, Muzi Na, Cindy W. Leung

**Affiliations:** 1 Department of Clinical Nutrition, Faculty of Applied Medical Sciences , King Abdulaziz University, 21589 Jeddah, Saudi Arabia; 2 Department of Nutritional Sciences, University of Michigan, 48109 Ann Arbor, MI USA; 3 Department of Nutritional Sciences, College of Health and Human Development, The Pennsylvania State University, University Park, Pennsylvania, USA; 4 Department of Nutrition, Harvard T.H. Chan School of Public Health, 02115 Boston, MA USA; 5 Department of Clinical Nutrition, Faculty of Applied Medical Sciences , King Abdulaziz University, 21589 Jeddah, Saudi Arabia

**Keywords:** Food insecurity, Quality of life, Perceived anxiety, Older adults, NHANES

## Abstract

**Background:**

Food insecurity is a significant health issue among older adults and contributes to poorer quality of life and mental health. However, limited evidence is available among older adults. Thus, the study evaluated the associations between food security and multiple outcomes related to health-related quality of life. We examined whether participants’ sex and participation in the federal Supplemental Nutrition Assistance Program (SNAP)/or receiving the Food Stamp program might modify these associations.

**Methods:**

Cross-sectional analysis of the 2007–2012 National Health and Nutrition Examination Surveys (NHANES). A sample of 3,375 adults aged ≥ 60 years with household incomes ≤ 300% of the federal poverty level (FPBL). Food security was assessed using the 18-item US Household Food Security Survey Module and categorized as food security, marginal food security, and food insecurity. Outcomes were the CDC Health-Related Quality of Life measures (HRQOL-4).

**Results:**

Approximately 8% experienced marginal food security and 12% experienced food insecurity. Over the past month, food insecurity was significantly associated with ≥ 16 days of poor physical health (OR 1.88, 95% CI 1.23, 2.85, *P*-trend = 0.005), ≥ 16 days of poor mental health (OR 2.22, 95% CI 1.50, 3.28, *P*-trend < 0.0001), and ≥ 16 days of feeling anxious (OR 3.33, 95% CI 2.30, 4.81, *P*-trend < 0.0001) after multivariate adjustment. The association between food insecurity and poor physical health was stronger in females (*P*-interaction = 0.02). There was no evidence for effect modification in any of these associations among those receiving benefits from the federal SNAP/Food Stamp program.

**Conclusions:**

Food insecurity was positively associated with multiple adverse health outcomes. Public health programs and policies targeted for older adults are needed to mitigate the extent of food insecurity to promote overall health and well-being.

## Background

In the US, there has been a documented increase in the population of older adults and their life expectancy over the last decade [1, 2]. In the next decade, older adults will represent 21% of the US population; their average life expectancy is estimated at 81.7 years in 2030, compared to 79.7 years in 2017 [1, 2]. Successful aging has been conceptualized as a descriptive measure for the quality of aging [3], aiming to reduce morbidity, optimize cognitive and functional capacities, and improve social engagement [4]. In particular, healthy nutrition is one of the core lifestyle approaches needed in public health programs to promote successful aging [5].

Food insecurity is a pressing public health concern for older adults [6–11]. In 2021, food insecurity was reported by 7.1% of US households with an adult ≥ 65 years and by 9.5% among adults ≥ 65 years living alone [12]. These figures reflect a substantial increase in food insecurity among older adults over the last 20 years [13]. Food insecurity disproportionately affects older adults who live alone, have fixed incomes, and suffer from chronic health conditions [6–11, 14]. Studies have shown that food insecurity is not only a critical risk factor for suboptimal dietary and health behaviors and physical health [15, 16], but also an important risk factor for poor mental health [17].

Previous studies have reported positive associations between food insecurity and adverse mental health outcomes [18–33]. These studies have mostly focused on younger-aged populations, including children, adolescents, and early to middle-aged adults [26, 34, 35]. Fewer studies have been conducted in the US older adults, and most of those have focused on depression as the primary outcome [19, 27, 28, 30–33]. In one study, the association between food insecurity and depression was significant among the sample of middle-older adults aged ≥ 51 years, but not among those aged ≥ 70 years [19]. Also, a limited number of studies have examined the link between food insecurity and health-related quality of life, with inconsistent findings [14, 36, 37]. Therefore, further studies are needed on the associations between food insecurity and health-related quality of life and mental health among older adults using a nationally representative sample.

Furthermore, the association between food insecurity and physical and mental health outcomes is complicated by the role of the Supplemental Nutrition Assistance Program (SNAP), formerly known as the Food Stamp Program. SNAP is the largest USDA food assistance program that provides low-income Americans with resources to purchase food. Although the primary goal is to reduce food insecurity, previous studies have been mixed on its ability to buffer the negative association between food insecurity and mental health among adults [19, 21, 28]. In one study, SNAP/Food Stamp program participation reduced the magnitude of the association between food insecurity and depression among low-income US adults aged ≥ 20 years [21]. In another study, food insecure US adults aged > 50 years receiving Food Stamp had a higher likelihood of depression compared to non-participating food insecure counterparts [28]. Further research is needed due to the inconsistency in findings from prior studies by evaluating the modulating the associations between food security and health-related quality of life.

The study aimed to evaluate the associations between household food security status and multiple outcomes related to health-related quality of life among older adults in the US aged ≥ 60 years. Also, we examined whether participants’ participation in SNAP/ Food Stamp program might modify the associations between food security and health outcomes. Lastly, because of the documented sex-related differences in the association between food insecurity and health outcomes [22, 38, 39], we evaluated the interaction between food insecurity by sex.

## Methods

### Study population

National Health and Nutrition Examination Survey (NHANES) NHANES is an ongoing, complex survey of a representative civilian, noninstitutionalized US population conducted by the National Center for Health Statistics. The Research Ethics Review Board at the National Center for Health Statistics approved the NHANES’s protocols. Written informed consent was collected from the participants or their proxies.

**Fig. 1:**
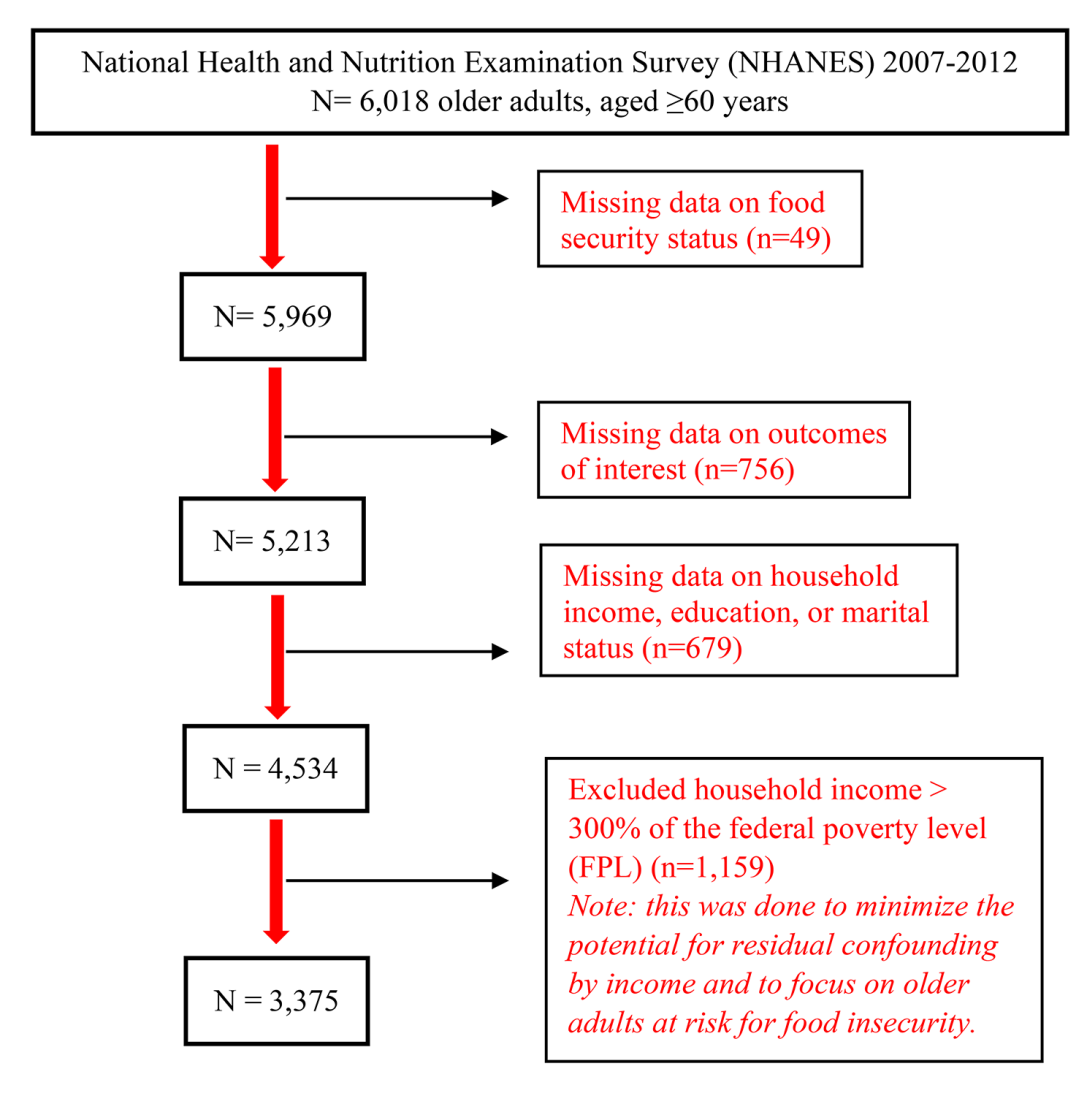
Flowchart of the exclusion criteria

Data from 2007 to 2012 were used because 2012 represents the final year when the CDC Health-Related Quality of Life measures were available in NHANES. The original study sample was 6,018 older adults, aged ≥ 60 years. Figure 1 shows the exclusion criteria and the final sample size.

### Household food security status

Household food security status was assessed in the NHANES household interview using the 18-item US Household Food Security Survey Module (HFSSM) by the US Department of Agriculture (USDA). The tool is the most widely used, validated measurement of household food security in the US In three stages, it assesses the experiences and behaviors of adults and children in the household related to insufficient resources to access food over the prior year. If there are no children present in the household, the eight questions pertaining to children’s experiences are omitted. According to USDA categories, the summed affirmative responses were categorized into: (1) food security (0 affirmative responses); (2) marginal food security (1–2 affirmative responses); and (3) food insecurity (≥ 3 affirmative responses) [40].

### Health-related quality of life

Mental health was assessed in the NHANES Mobile Examination Center using a validated instrument, called the CDC Health-Related Quality of Life (HRQOL-4) Healthy Days core questions. Three questions from the HRQOL-4 pertain to self-reported days in the past 30 days when: (1) one’s physical health was “not good,” (2) one’s mental health was “not good,” and (3) one felt “worried, tense, or anxious.” Responses to these three questions were categorized into 0 days, 1–5 days, 6–15 days, and 16–30 days [41].

### Study covariates

Study covariates were selected a priori based on known associations between these variables with food insecurity and health-related quality of life and depression outcomes. Sociodemographic covariates included participant’s age (60–64, 65–69, 70–74, 75–79, ≥ 80), sex (male, female), race and ethnicity (Non-Hispanic White, Non-Hispanic Black, Hispanic, or “Other” racial group), level of education (< 12 years, high school diploma/equivalent, some college, or college graduate), marital status (married/living with a partner, never married, separated/divorced/widowed), and poverty income ratio (0–100% FPL, > 100–130% FPL, > 130–200% FPL, > 200–250% FPL, > 250–300% FPL). Participants also reported whether their household received any SNAP or Food Stamp program benefits over the past year (yes/no).

### Statistical analysis

All statistical analyses incorporated NHANES complex survey weights recalculated to reflect the six-year period, to address the unequal selection probabilities and patterns of non-response across the different NHANES components, and to yield nationally representative estimates. First, descriptive analyses were conducted for sociodemographic variables and mental health outcomes for the total older adult sample and stratified by food security categories. Differences in descriptive characteristics by food security categories were tested using Chi-square tests. The associations between household food security and health-related quality of life were examined using multivariate multinomial logistic regression models. Models were adjusted for sociodemographic factors and the NHANES survey cycle. Tests for trends were conducted by modeling food security status as an ordinal variable in the multivariate models. We further explored the potential of sex and participation in the SNAP/Food Stamp program to modify the main associations of interest in testing the joint significance of cross-product terms between sex or SNAP participation and food security categories. All statistical tests were 2-sided, and statistical significance was considered at *P* < 0.05. Statistical analyses were performed using SAS 9.4 (SAS Institute, Cary, NC).

## Results

Approximately 8% of older adults experienced marginal food security and 12% of older adults experienced food insecurity in the past 12 months. Table 1 shows descriptive characteristics by food security status. Compared to adults aged 70 and higher, adults aged 60–64 years were more likely to have marginal food security and food insecurity (*P* < 0.0001). Non-Hispanic Black and Hispanic older adults were more likely to have marginal food security and food insecurity compared to the Non-Hispanic White group (*P* < 0.0001). Older adults with lesser years of education were likely to experience marginal food security or food insecurity (*P* < 0.0001). Similarly, marginal food security and food insecurity were more prevalent among those with lower household incomes (*P* < 0.0001). And finally, older adults who were separated, widowed, or divorced were more likely to have marginal food security and food insecurity when compared to those who were married or living with a partner (*P* < 0.0001). There was no difference in food security status by sex (*P =* 0.55). In bivariate analyses, greater severity of food insecurity was associated with more days of poor physical health, poor mental health and feeling worried, tense, or anxious (*P*s < 0.0001).

**Table 1:** Descriptive characteristics by household food security status among US older adults attending National Health and Nutrition Examination Survey (NHANES) 2007–2012

	Total *n* = 3,375	Food security *n* = 2,424	Marginal food security *n* = 385	Food insecurity *n* = 566	*P*-value
	n (%)	n (%)	n (%)	n (%)	
Age (years)					< 0.0001^*^
60–64	862 (23.8)	512 (21.3)	135 (31.7)	215 (35.1)	
65–69	676 (19.8)	450 (18.9)	83 (20.3)	143 (26.0)	
70–74	629 (18.7)	468 (19)	79 (22.4)	82 (13.9)	
75–79	502 (14.7)	391 (15.4)	47 (11.8)	64 (11.7)	
≥80	706 (23.0)	603 (25.4)	41 (13.7)	62 (13.3)	
Sex					0.55
Male	1599 (40.5)	1160 (40.9)	180 (38.7)	259 (39.6)	
Female	1776 (59.5)	1264 (59.1)	205 (61.3)	307 (60.4)	
Race/ethnicity					< 0.0001^*^
Non-Hispanic White	1598 (72.9)	1335 (78.7)	114 (51.4)	149 (47.7)	
Non-Hispanic Black	754 (11.3)	484 (9)	116 (22.3)	154 (19.5)	
Hispanic	821 (10.3)	468 (7.3)	130 (20.4)	223 (23.4)	
Other	202 (5.6)	137 (5)	25 (5.9)	40 (9.4)	
Education					< 0.0001^*^
<12 years	1501 (34.6)	958 (30.2)	202 (46.7)	341 (56.2)	
High school diploma or equivalent	855 (30.4)	659 (32.4)	78 (23.9)	118 (21.5)	
Some college	702 (24.0)	536 (25.1)	79 (20.6)	87 (18.5)	
College graduate	317 (11.0)	271 (12.3)	26 (8.8)	20 (3.8)	
Marital status					< 0.0001^*^
Married or living with a partner	1715 (54.5)	1305 (57.5)	173 (45.1)	237 (40.5)	
Never married	186 (4.4)	119 (4.2)	28 (6.4)	39 (5.1)	
Separated, widowed, or divorced	1474 (41.1)	1000 (38.3)	184 (48.5)	290 (54.4)	
SNAP participation (< 130% FPL)					< 0.0001^*^
Non-participant	2708 (85.8)	2130 (91.6)	243 (63.3)	335 (61.2)	
SNAP participant	664 (14.2)	93 (8.4)	140 (36.7)	231 (38.8)	
Poverty income ratio					< 0.0001^*^
0–100% FPL	921 (19.3)	501 (14.1)	152 (36.3)	268 (43.3)	
>100–130% FPL	622 (16.0)	404 (14.2)	82 (18.9)	136 (25.7)	
>130–200% FPL	911 (28.9)	716 (30.7)	84 (3.2)	111 (20.3)	
>200–250% FPL	531 (20.3)	451 (23.0)	51 (14.2)	29 (5.6)	
>250–300% FPL	390 (15.6)	352 (18.0)	16 (7.4)	22 (5.2)	
Days of poor physical health/ month					< 0.0001^*^
0 days	1882 (61.2)	1448 (64.3)	201 (59.2)	233 (41.1)	
1–5 days	435 (15.1)	286 (14.4)	45 (13.4)	104 (21.4)	
6–15 days	343 (10.7)	228 (9.8)	43 (11.2)	72 (15.4)	
16–30 days	447 (13)	278 (11.5)	53 (15.2)	116 (22.1)	
Days of poor mental health/ month					< 0.0001^*^
0 days	2180 (68.6)	1642 (70.6)	221 (61.0)	317 (59.7)	
1–5 days	439 (15.3)	313 (15.9)	54 (16.1)	72 (11.1)	
6–15 days	229 (7.7)	145 (6.9)	26 (9.8)	58 (12.2)	
16–30 days	262 (8.4)	143 (6.7)	42 (13.0)	77 (17.0)	
Days felt worried, tense, or anxious/ month					< 0.0001^*^
0 days	1706 (54.0)	1339 (57.4)	165 (46.4)	202 (35.2)	
1–5 days	706 (23.6)	486 (23.1)	89 (27.7)	131 (23.8)	
6–15 days	324 (10.7)	208 (9.8)	35 (10.9)	81 (16.7)	
16–30 days	371 (11.8)	209 (9.7)	53 (15.0)	109 (24.3)	

SNAP = Supplemental Nutrition Assistance Program; FPL = Federal Poverty Level

^*^P-value < 0.001

Table 2 shows the associations between food security status and health-related quality of life outcomes among older adults. For each health domain, greater severity of food insecurity was associated with greater days of poor health after adjustment for sociodemographic characteristics (*P*-trends < 0.05). For physical health, food-insecure adults were more likely to have 1–5 days (OR 1.98, 95% CI 1.39, 2.83, *P*-trend = 0.0007), 6–15 days (OR 1.91, 95% CI 1.21, 3.02, 0.009, *P*-trend = 0.009), and 16–30 days (OR 1.88, 95% CI 1.23, 2.85, *P*-trend = 0.005) of reporting poor physical health compared to food-secure adults. For mental health, food-insecure adults were more likely to have 6–15 days (OR 1.85, 95% CI 1.14, 3.00, *P*-trend = 0.01), and 16–30 days (OR 2.22, 95% CI 1.50, 3.28, *P*-trend < 0.0001) of reporting poor mental health compared to food-secure adults. For feelings of worry and anxiety, food-insecure older adults were more likely to have 1–5 days (OR 1.72, 95% CI 1.28, 2.31, *P*-trend = 0.0001), 6–15 days (OR 2.78, 95% CI 1.94, 4.00, *P*-trend < 0.0001), and 16–30 days (OR 3.33, 95% CI 2.30, 4.81, *P*-trend < 0.0001) of reporting feeling worried, tense, or anxious, compared to food-secure adults. Moreover, marginal food insecure older adults were more likely to have 16–30 days (OR = 1.77, 95% CI 1.03, 3.02, *P*-value < 0.05) of reporting poor mental health/ month and feeling worried, tense, or anxious (OR = 1.62, 95% CI 1.09, 2.40, *P*-value < 0.05), compared to food-secure adults.

**Table 2:** Multivariate-adjusted associations ^a^ between food security status and health-related quality of life among US older adults attending National Health and Nutrition Examination Survey (NHANES) 2007–2012

	Food security n= 2,424	Marginal food security *n* = 385		Food insecurity *n* = 566		P-trend	P-interaction	
							SNAP/ food stamp	sex
	OR	OR 95% CI	*P*-value	OR 95% CI	*P*-value			
Days of poor physical health/ month							0.28	0.02*
0 days (Ref.)								
1–5 days	Ref.	0.96 (0.67, 1.38)	0.82	1.98 (1.39, 2.83) *	0.0002**	0.0007**		
6–15 days	Ref.	1.06 (0.67, 1.67)	0.81	1.91 (1.21, 3.02) *	0.005*	0.009**		
16–30 days	Ref.	1.23 (0.82, 1.84)	0.32	1.88 (1.23, 2.85) *	0.003*	0.005**		
Days of poor mental health/ month							0.3	0.35
0 days (Ref.)								
1–5 days	Ref.	1.25 (0.86, 1.82)	0.25	0.86 (0.56, 1.33)	0.50	0.75		
6–15 days	Ref.	1.51 (0.87, 2.64)	0.15	1.85 (1.14, 3.00) *	0.01*	0.01*		
16–30 days	Ref.	1.77 (1.03, 3.02) *	0.04*	2.22 (1.50, 3.28) *	< 0.0001**	< 0.0001**		
Days felt worried, tense, or anxious/ month							0.81	0.34
0 days (Ref.)								
1–5 days	Ref.	1.41 (0.99, 2.01)	0.06	1.72 (1.28, 2.31) *	0.0003**	0.0001**		
6–15 days	Ref.	1.32 (0.77, 2.24)	0.31	2.78 (1.94, 4.00) *	< 0.0001**	< 0.0001**		
16–30 days	Ref.	1.62 (1.09, 2.40) *	0.02*	3.33 (2.30, 4.81) *	< 0.0001**	< 0.0001**		

^a^ Models were adjusted for age, sex, race/ethnicity, education, marital status, poverty: income ratio, and survey year

* *P*-value < 0.05

** *P*-value < 0.001

There was no evidence that SNAP/Food Stamp program benefits participation modified the associations between household food security status and any of the mental health indicators (*Ps*-interactions > 0.2) (Table 2). Because the interaction with sex was statistically significant for poor physical health **(***P*- interaction = 0.02) in Tables 2 and 3 shows a sex-stratified analysis for the associations between food security status and poor physical health. For older adult females, food insecurity was associated with elevated odds of 1–5 days (OR 2.46, 95% CI 1.63, 3.71, *P*-trend = 0.0004), 6–15 days (OR 1.78, 95% CI 1.08, 2.96, *P*-trend = 0.03), and 16–30 days (OR 2.39, 95% CI 1.50, 3.81, *P*-trend = 0.0001) of poor physical health. For older adult males, food insecurity was associated with elevated odds of 6–15 days of poor physical health (OR 2.31, 95% CI 1.11, 4.84, *P*-trend = 0.04), but associations between food insecurity and 1–5 days or 16–30 days of poor physical health were not significant.

**Table 3:** Sex-stratified multivariate-adjusted associations ^a^ between food security status and health-related quality of life among US older adults attending National Health and Nutrition Examination Survey (NHANES) 2007–2012

	Food security *n* = 2,424	Marginal food security *n* = 385		Food insecurity *n* = 566		P-trend
	OR	OR 95% CI	*P*-value	OR 95% CI	*P*-value	
Days of poor physical health/ month						
Males						
0 days (Ref.)						
1–5 days	Ref.	1.18 (0.55, 2.54)	0.66	1.39 (0.70, 2.77)	0.35	0.36
6–15 days	Ref.	1.03 (0.45, 2.38)	0.94	2.31 (1.11, 4.84) *	0.03	0.04 *
16–30 days	Ref.	1.00 (0.56, 1.78)	0.99	1.40 (0.81, 2.42)	0.23	0.28
Females						
0 days (Ref.)						
1–5 days	Ref.	0.84 (0.51, 1.37)	0.48	2.46 (1.63, 3.71) *	< 0.0001**	0.0004 **
6–15 days	Ref.	1.06 (0.59, 1.91)	0.85	1.78 (1.08, 2.96) *	0.03*	0.03 *
16–30 days	Ref.	1.40 (0.84, 2.32)	0.20	2.39 (1.50, 3.81) *	0.0002**	0.0001 **

^a^ Models were adjusted for age, race/ethnicity, education, marital status, poverty: income ratio, and survey year

* *P*-value < 0.05

** *P*-value < 0.001

## Discussion

Using a nationally representative sample of older adults (≥ 60 years) in the US, our results showed that approximately one in five lower-income older adults experienced marginal food security and food insecurity at some point during the past year. Our results also demonstrate the strong associations between food insecurity and poorer health-related quality of life, independent of other sociodemographic correlates. The majority of these associations showed a consistent dose-response relationship, with greater severity of food insecurity associated with poorer health outcomes.

The significant associations between food insecurity and poorer health-related quality of life align with previous observational studies conducted in the US that linked food insecurity with a decline in health-related quality of life among adults aged 50–80 years [14], and adults aged ≥ 60 years [36]. Moreover, food insecurity has also been associated with poor diet quality [8, 33, 36, 42], and other age-related outcomes (e.g., functional limitations [42], chronic diseases [10, 14, 29], and cognitive impairment [16]). Poor self-rated physical and mental health could explain some of these observed associations between food insecurity and health outcomes in older adults. For example, Jung et al. showed that depression mediated the association between food insecurity and poor nutritional status among low-income US adults aged > 60 years [27]. Longitudinal studies are needed to understand how the perceived health-related quality of life and mental health may contribute to the chronic health implications of food insecurity among older adults.

Our results further showed that even older adults with marginal food security had significantly elevated odds of experiencing poor mental health and anxiety at least half of the month. This evidence highlights that even marginal food security is associated with self-rated poor mental health and anxiety, which corroborates previous research showed that marginal food security or mild food insecurity, was associated with an impaired cardiometabolic profile [43, 44], and lower diet quality [8]. During the early days of the COVID-19 pandemic, marginal food security was associated with higher odds of depression, anxiety, and high perceived stress among US adults [45]. Therefore, additional research is needed to understand the psychosocial and behavioral consequences on older adults experiencing marginal food security, to help explain these associations with a mild form of insecurity.

We showed that food-insecure older females were more likely to have self-rated poor physical health compared to food-insecure older males. Our results are similar to prior findings indicating differences in the association between food insecurity and health outcomes by sex [22, 38, 39, 46]. Previous studies showed that food insecurity was associated with higher anxiety [39], psychological distress [38], levels of stress [22], and obesity [46] among females. On the other hand, some evidence showed that food insecurity was associated with higher odds of depression among men compared to women [35], while others showed a lack of sex difference on the impact of food insecurity on mental health [21]. Given the scarcity of evidence that investigated the sex difference in the impact of food insecurity on health, and the potential of residual socioeconomic confounding by sex in observational studies [38], further studies are needed to evaluate the sex difference in the association between food insecurity and health outcomes.

We found no significant differences by participation in the SNAP/Food Stamp program on the associations between food security status and health-related quality of life, which may be due to the low participation rate among our study population. Our results showed that only 38.8% of older adults with food insecurity participated in the program, which corroborates recent evidence that eligible older adults have one of the lowest program participation rates across all age groups [47]. In addition, older adults may qualify for lower SNAP benefit allotments (due to smaller household sizes), may participate for variable durations, and may also receive benefits from other health and economic programs (e.g., Medicare, social security). All of these reasons may have tempered the ability of SNAP to modify the associations between food security status and health-related quality of life observed in the present study. Our results on the lack of a statistical difference between SNAP participants and non-participants are consistent with prior null findings in SNAP/Food Stamp to buffer the negative effect of food insecurity on depression [19, 28], and diet quality [8] among middle-older adults. However, other studies showed favorable impacts from the SNAP/Food Stamp program in mitigating food insecurity consequences on mental health [19, 21]. It is worth noting that the participation in the Food Stamp program was associated with lower self-esteem and negative self-attitudes [28], and higher emotional distress [48], which was attributed to stigma surrounding program use [48]. While it is widely recognized that SNAP alleviates food insecurity in the US population, additional outreach efforts are needed to increase participation among income-eligible older adults [27] in the community and clinical settings, and further work is needed to eliminate the stigma for participating in SNAP or other food and economic relief programs in the US. Furthermore, we examined the potential role of only one of the federally funded programs for older adults collected in the NHANES, SNAP/Food Stamp. Because of the existence of other federally funded programs for older adults, such as the Older Americans Act [49], further studies should examine the role of other health programs for older adults in influencing the association between food insecurity and health outcomes among older adults.

This study has some limitations. First, due to the cross-sectional nature of the data, we are unable to infer any causal relationships between food insecurity, health-related quality of life among older adults. Even though household food security status was assessed over the past year and health outcomes were reported over the past month, reverse causation remains plausible because some of the outcomes we examined may also influence food security status [24]. Longitudinal studies are needed to understand the direction of the association among older adults. Second, the possibility of unmeasured confounding could not be ruled out. Factors like transportation needs, neighborhood access to food stores, household assets to purchase food other than income, and social support were not measured in NHANES.

Another limitation is that the USDA HFSSM was developed primarily from research with caregivers with young children as the reference. Older adults may face unique barriers to food acquisition (e.g., functional limitations, health conditions, social isolation) that are not captured in the current measure, which primarily focuses on economic restraints. While the HFSSM is the gold standard for food insecurity assessment in the US context, the prevalence of true food insecurity in the study population may be underestimated. We acknowledge that we examined the potential role of SNAP/Food Stamp, which is only one of the federally funded programs for older adults in the US collected in the NHANES; therefore, our findings could not be extrapolated to other federally funded programs, such as the Older Americans Act [49]. Thus, further studies should examine the role of other health and nutrition programs in influencing the association between food insecurity and health outcomes among older adults.

Moreover, because of the small sample, we were limited from examining all four groups of food security (i.e., food security, marginal food security, low food security, and very low food security); thus, future studies with larger sample sizes to examine the dose-response nature of these associations across the four groups of food security are warranted. Also, due to some degree of sensitivity in reporting household food security information and mental health, we acknowledge that our self-reported data might be subject to social desirability bias. However, this would report an under-reporting of food insecurity and poorer physical and mental health outcomes, which would suggest that the reported estimates are attenuated compared to the true estimates. Further studies are needed to confirm these associations among older adults in different geographic and socio-political contexts.

## Conclusions

In conclusion, we showed that food insecurity was associated with poorer health-related quality of life among US older adults aged ≥ 60 years. To a lesser extent, positive associations were also observed between marginal food security and some health-related quality of life outcomes. There was some evidence of heterogeneity in these associations by sex, but not by participation in the SNAP/Food Stamp program. Further research is needed to examine the underlying mechanisms linking food insecurity and the physical and mental health outcomes observed in the current study and explore the potential of diet quality and other modifiable risk factors to ameliorate the adverse associations between food insecurity and poor mental health among older adults.

## Data Availability

The datasets generated and/or analyzed during the current study are available in the National Health and Nutrition Examination Survey repository, [https://www.cdc.gov/nchs/nhanes/index.htm].

## Abbreviations

SNAP: Supplemental Nutrition Assistance Program
NHANES: National Health and Nutrition Examination Survey
FPL: Federal Poverty Level
HRQOL-4: Health-Related Quality of Life
